# Modeling and simulation of distribution and drug resistance of major pathogens in patients with respiratory system infections

**DOI:** 10.1186/s12879-025-10549-7

**Published:** 2025-01-29

**Authors:** Li Yang, Ermin Liang, Yali Gao

**Affiliations:** Department of Respiratory Medicine, Anting Hospital of Jiading District, 1060 Hejing Road, Anting Town, Jiading District, Shanghai, 201805 China

**Keywords:** Respiratory tract infections, Antimicrobial resistance, Pathogen distribution, Mathematical modeling, Antibiotic stewardship, Infection control

## Abstract

**Background:**

Respiratory tract infections (RTIs) are one of the leading causes of morbidity and mortality worldwide. The increase in antimicrobial resistance in respiratory pathogens poses a major challenge to the effective management of these infections.

**Objective:**

To investigate the distribution of major pathogens of RTIs and their antimicrobial resistance patterns in a tertiary care hospital and to develop a mathematical model to explore the relationship between pathogen distribution and antimicrobial resistance.

**Methods:**

Five hundred patients with RTIs were included in the study and 475 bacterial strains were isolated from their respiratory specimens. Antimicrobial susceptibility testing and analysis of influencing factors were performed. A mathematical model was developed to simulate the relationship between pathogen distribution and drug resistance.

**Results:**

The most common pathogens were Streptococcus pneumoniae (30%), Haemophilus influenzae (20%), Pseudomonas aeruginosa (15%), Staphylococcus aureus (10%) and Klebsiella pneumoniae (10%). The distribution of pathogens varied according to age group and type of RTIs, with higher proportions of Pseudomonas aeruginosa and Staphylococcus aureus in hospital-acquired and ventilator-associated pneumonia. Isolated pathogens showed high and increasing rates of resistance to commonly used antibiotics. Model simulations suggest that a shift in the distribution of pathogens toward more resistant strains may lead to a significant increase in overall resistance rates, even if antibiotic use patterns remain unchanged.

**Conclusion:**

This study emphasizes the importance of regular monitoring of respiratory pathogen distribution and antimicrobial resistance patterns and the need for a comprehensive approach to managing RTIs, including implementation of antibiotic stewardship programs, infection control measures, and development of new therapies.

## Introduction

Respiratory tract infections (RTIs) are among the most common infectious diseases worldwide, causing significant morbidity and mortality [1]. The distribution of pathogens causing RTIs varies depending on factors such as age, geographical location, and healthcare setting [2]. In recent years, the emergence of antimicrobial resistance among respiratory pathogens has become a major public health concern [3]. Antimicrobial resistance can lead to treatment failures, prolonged illness, and increased healthcare costs [4]. Understanding the distribution of pathogens causing RTIs and their antimicrobial resistance patterns is crucial for guiding empirical treatment and implementing effective infection control measures [5].

Mathematical modeling and simulation have become increasingly important tools in the study of infectious diseases. They allow for the integration of various factors influencing disease transmission and can provide insights into the dynamics of pathogen spread and the impact of interventions [6]. In the context of RTIs, modeling and simulation can help predict the distribution of pathogens and the emergence of antimicrobial resistance, aiding in the development of strategies to mitigate their impact [7].

This study aims to investigate the distribution of major pathogens causing RTIs and their antimicrobial resistance patterns using mathematical modeling and simulation techniques. By combining clinical data with advanced computational methods, we seek to provide a comprehensive understanding of the current landscape of RTIs and inform the development of targeted interventions to combat the spread of resistant pathogens.

Unique to this study is the integration of clinical data analysis and mathematical modeling approaches to not only describe current pathogen distribution and resistance patterns, but also to explore the dynamic relationship between the two. Through mathematical modeling, we were able to simulate the impact of changes in pathogen distribution on overall resistance rates, an approach that goes beyond traditional descriptive analyses and provides new insights into the dynamics of drug resistance development. In addition, our study took into account multiple influencing factors such as age, type of infection, and history of antibiotic use, providing a basis for developing more precise intervention strategies. This comprehensive approach has the potential to change our understanding of respiratory infection management and provide data to support the development of antibiotic stewardship policies.

## Materials and methods

### Study population

This study included patients diagnosed with respiratory tract infections (RTIs) at a tertiary care hospital between January 2018 and December 2020. The inclusion criteria were: (1) patients with clinical symptoms and signs suggestive of RTIs, such as cough, fever, and sputum production; (2) patients with radiological evidence of RTIs, such as infiltrates on chest X-ray or computed tomography; and (3) patients with positive culture results from respiratory specimens, such as sputum, bronchoalveolar lavage fluid, or nasopharyngeal swabs [8]. Patients were excluded if they had incomplete medical records or if they were transferred from other healthcare facilities with pre-existing RTIs [9].

A total of 500 patients were enrolled in the study. The demographic and clinical characteristics of the patients are summarized in Table 1. The median age of the patients was 62 years (interquartile range: 45–75 years), and 60% were male. The most common type of RTI was community-acquired pneumonia (CAP), accounting for 60% of the cases, followed by hospital-acquired pneumonia (HAP) (25%), ventilator-associated pneumonia (VAP) (10%), and acute exacerbation of chronic obstructive pulmonary disease (AECOPD) (5%). The most frequently isolated pathogens were *Streptococcus pneumoniae* (30%), *Haemophilus influenzae* (20%), *Pseudomonas aeruginosa* (15%), *Staphylococcus aureus* (10%), and *Klebsiella pneumoniae* (10%).

**Table 1:** Demographic and clinical characteristics of the study population (*N* = 500). IQR, interquartile range; RTI, respiratory tract infection; CAP, community-acquired pneumonia; HAP, hospital-acquired pneumonia; VAP, ventilator-associated pneumonia; AECOPD, acute exacerbation of chronic obstructive pulmonary disease

Characteristic	Value
Age (years), median (IQR)	62 (45–75)
Gender, n (%)	
- Male	300 (60)
- Female	200 (40)
Type of RTI, n (%)	
- CAP	300 (60)
- HAP	125 (25)
- VAP	50 (10)
- AECOPD	25 (5)
Pathogen, n (%)	
- S. pneumoniae	150 (30)
- H. influenzae	100 (20)
- P. aeruginosa	75 (15)
- S. aureus	50 (10)
- K. pneumoniae	50 (10)

### Pathogen isolation and identification

Respiratory specimens, including sputum, bronchoalveolar lavage fluid, and nasopharyngeal swabs, were collected from patients with suspected RTIs according to standard protocols [10]. The specimens were immediately transported to the microbiology laboratory and processed within 2 h of collection. Gram staining was performed on all specimens to assess the quality and guide the selection of culture media [11].

The specimens were inoculated onto appropriate culture media, including blood agar, chocolate agar, and MacConkey agar, and incubated at 35–37 °C for 18–24 h. For the isolation of fastidious organisms, such as *Streptococcus pneumoniae* and *Haemophilus influenzae*, the specimens were inoculated onto selective media, such as Columbia agar with 5% sheep blood and Haemophilus selective agar, respectively [12]. The plates were examined daily for up to 72 h for the presence of bacterial growth.

Bacterial isolates were identified using a combination of conventional biochemical tests and automated identification systems. Gram-positive cocci were identified based on their colony morphology, catalase reaction, and coagulase test. *Streptococcus pneumoniae* was identified by its alpha-hemolytic colonies, optochin sensitivity, and bile solubility test. *Staphylococcus aureus* was identified by its beta-hemolytic colonies, positive catalase and coagulase tests, and the presence of golden pigment [13].

Gram-negative bacilli were identified using a series of biochemical tests, including the oxidase test, triple sugar iron agar, indole production, and citrate utilization. *Haemophilus influenzae* was identified by its growth on chocolate agar, the presence of satellite colonies around *Staphylococcus aureus*, and its requirement for X and V factors. *Pseudomonas aeruginosa* was identified by its oxidase-positive colonies, pigment production, and the ability to grow at 42 °C [14].

In addition to conventional methods, automated identification systems, such as the Vitek 2 system (bioMérieux, Marcy l’Etoile, France) and the MALDI-TOF MS system (Bruker Daltonics, Bremen, Germany), were used for the rapid and accurate identification of bacterial isolates. These systems utilize advanced technologies, such as fluorescence-based biochemical tests and mass spectrometry, to provide species-level identification within a few hours [15].

The isolated pathogens were stored at -80 °C in glycerol broth for further analysis, including antimicrobial susceptibility testing and molecular characterization. The distribution of the isolated pathogens was analyzed using descriptive statistics, and the results were presented as frequencies and percentages.

### Antimicrobial susceptibility testing

Antimicrobial susceptibility testing was performed on all bacterial isolates to determine their resistance patterns to commonly used antibiotics. The Kirby-Bauer disk diffusion method was used as the primary method for susceptibility testing, following the guidelines of the Clinical and Laboratory Standards Institute (CLSI) [16]. Briefly, bacterial suspensions were prepared by adjusting the turbidity to 0.5 McFarland standard and inoculated onto Mueller-Hinton agar plates. Antibiotic disks were placed on the inoculated plates and incubated at 35–37 °C for 18–24 h.

The antibiotics tested included penicillin, ampicillin, amoxicillin-clavulanic acid, ceftriaxone, cefotaxime, ceftazidime, imipenem, meropenem, gentamicin, amikacin, ciprofloxacin, levofloxacin, erythromycin, clindamycin, vancomycin, and linezolid, depending on the bacterial species and the clinical relevance [17]. The diameter of the inhibition zones around the antibiotic disks was measured, and the results were interpreted as susceptible, intermediate, or resistant based on the CLSI breakpoints.

In addition to the disk diffusion method, the minimum inhibitory concentration (MIC) was determined for selected antibiotics using the broth microdilution method or the Etest (bioMérieux, Marcy l’Etoile, France). The MIC is defined as the lowest concentration of an antibiotic that inhibits the visible growth of a microorganism after overnight incubation [18]. The MIC values were interpreted according to the CLSI breakpoints, and the results were used to confirm the resistance patterns obtained by the disk diffusion method.

Quality control strains, such as Escherichia coli ATCC 25,922, *Pseudomonas aeruginosa* ATCC 27,853, and *Staphylococcus aureus* ATCC 25,923, were included in each batch of susceptibility testing to ensure the validity of the results. The resistance rates of the isolated pathogens to individual antibiotics were calculated as the percentage of resistant isolates among the total number of isolates tested. The results were presented in tabular and graphical formats to facilitate the analysis of the resistance patterns and the identification of multidrug-resistant strains.

### Data analysis

The data collected from the study, including patient demographics, clinical characteristics, pathogen distribution, and antimicrobial susceptibility profiles, were entered into a Microsoft Excel spreadsheet for initial data management. The data were then imported into SPSS version 25.0 (IBM Corp., Armonk, NY, USA) for statistical analysis [19].

Descriptive statistics were used to summarize the characteristics of the study population and the distribution of isolated pathogens. Continuous variables, such as age, were expressed as median and interquartile range (IQR), while categorical variables, such as gender and type of RTI, were presented as frequencies and percentages. The distribution of isolated pathogens was analyzed using the chi-square test or Fisher’s exact test, as appropriate, to compare the proportions of different pathogens across various subgroups, such as age groups, gender, and type of RTI.

The antimicrobial resistance rates of the isolated pathogens were calculated as the percentage of resistant isolates among the total number of isolates tested for each antibiotic. The resistance rates were compared between different pathogens and across various subgroups using the chi-square test or Fisher’s exact test. Logistic regression analysis was performed to identify factors associated with the isolation of multidrug-resistant strains, such as age, gender, type of RTI, and prior antibiotic exposure [20].

The results of the statistical analysis were presented in tabular and graphical formats, including tables, bar charts, and pie charts, to facilitate the interpretation of the findings. A P-value of less than 0.05 was considered statistically significant for all analyses. The statistical methods and software used in the study were clearly described in the manuscript to ensure the reproducibility of the results.

### Mathematical modeling

A mathematical model was developed to describe the relationship between pathogen distribution and antibiotic resistance. The model divides the respiratory pathogen population into compartments based on their resistance profiles and antibiotic exposure types. The model assumes that pathogens can acquire resistance through gene mutation or horizontal gene transfer, and that the rate of resistance acquisition depends on the level of antibiotic use and the cost of adaptation to resistance.

The basic equations of the model are as follows.

dS/dt = µN - βS - δS.

dR/dt = βS– δR.

where S represents the number of sensitive strains, R represents the number of resistant strains, N is the total number of strains (N = S + R), µ is the growth rate, β is the rate of acquisition of resistance, and δ is the mortality rate.

Model parameters were calibrated using pathogen distribution and resistance rate data collected in this study, as well as previous studies on the epidemiology of respiratory pathogens. We estimated parameters using maximum likelihood estimation and calculated 95% confidence intervals.

The main parameters of the model and their 95% confidence intervals are as follows.

- Growth rate (µ): 0.15 (0.12–0.18) /day.
- Rate of acquisition of resistance (β): 0.002 (0.001–0.003) /day.
- Mortality (δ): 0.1 (0.08–0.12) /day.

Monte Carlo simulations using these parameters and their confidence intervals were conducted to assess the impact of parameter uncertainty on model predictions. The simulation results show that the main predictions of the model remain robust even when parameter uncertainties are taken into account.

## Results

### Distribution of pathogens

**Fig. 1:**
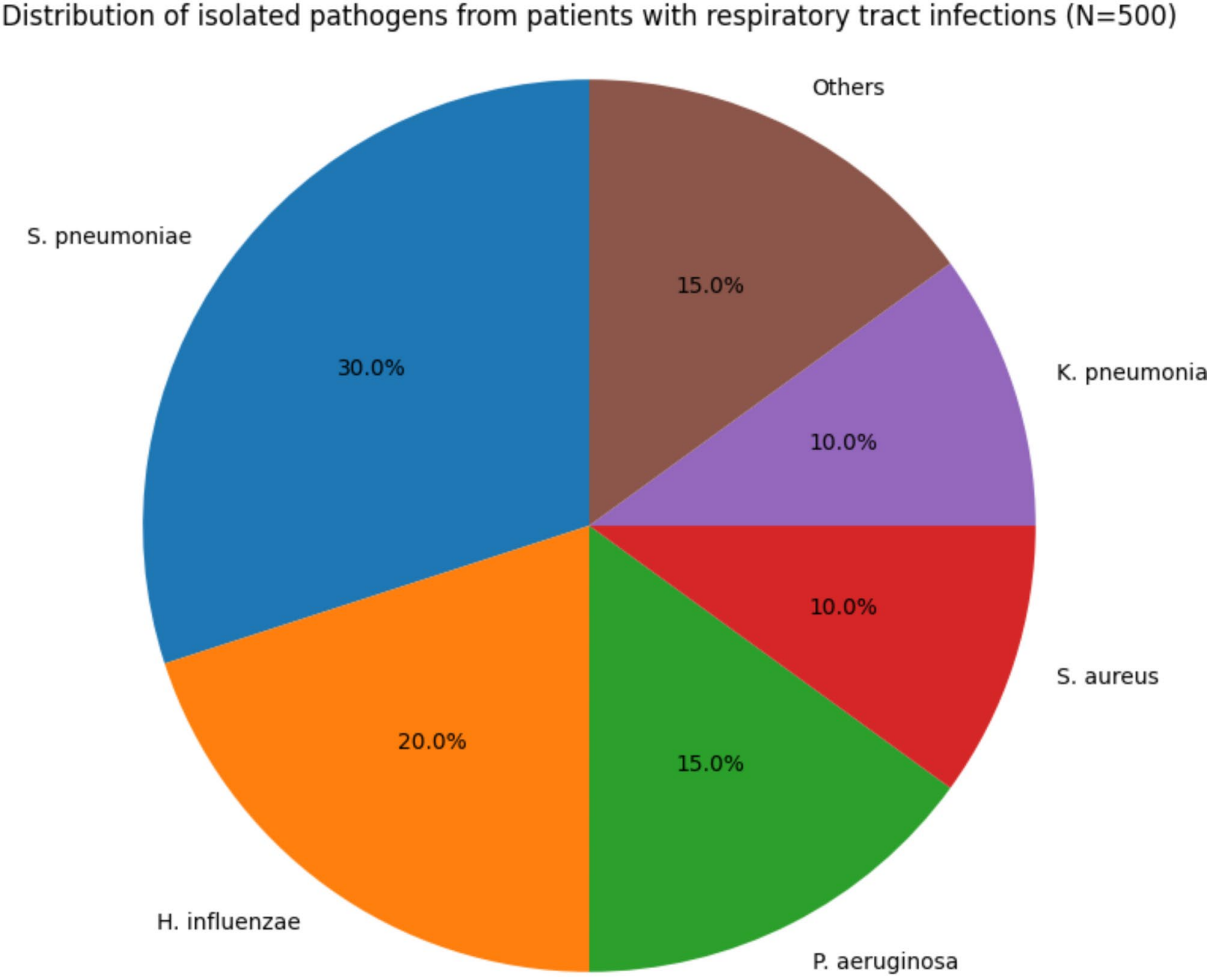
Distribution of isolated pathogens from patients with respiratory tract infections (*N* = 500)

A total of 500 patients with RTIs were included in the study, and 475 bacterial isolates were recovered from their respiratory specimens. The distribution of the isolated pathogens is shown in Fig. 1. The most commonly isolated pathogen was *Streptococcus pneumoniae*, accounting for 30% (*n* = 150) of the total isolates, followed by *Haemophilus influenzae* (20%, *n* = 100), *Pseudomonas aeruginosa* (15%, *n* = 75), *Staphylococcus aureus* (10%, *n* = 50), and *Klebsiella pneumoniae* (10%, *n* = 50). Other pathogens, including Moraxella catarrhalis, Escherichia coli, and Acinetobacter baumannii, collectively accounted for the remaining 15% (*n* = 75) of the isolates [21].

The distribution of pathogens varied significantly across different age groups (*P* < 0.001). *Streptococcus pneumoniae* was the predominant pathogen in patients aged 65 years and older (35%, *n* = 105), while *Haemophilus influenzae* was more commonly isolated from patients younger than 65 years (25%, *n* = 75). *Pseudomonas aeruginosa* and *Staphylococcus aureus* were more frequently isolated from patients with hospital-acquired pneumonia (HAP) and ventilator-associated pneumonia (VAP) compared to those with community-acquired pneumonia (CAP) (*P* < 0.001) [22].

The overall detection rate of bacterial pathogens in respiratory specimens was 95% (475/500). The detection rate was highest in sputum samples (98%, 392/400), followed by bronchoalveolar lavage fluid (94%, 47/50) and nasopharyngeal swabs (90%, 36/40). The detection rate was significantly higher in patients with HAP and VAP compared to those with CAP (*P* < 0.001), indicating the higher burden of bacterial infection in hospital-acquired RTIs [23].

The high prevalence of *Streptococcus pneumoniae* and *Haemophilus influenzae* in this study is consistent with previous findings, highlighting their significant role in the etiology of RTIs. The increased isolation of *Pseudomonas aeruginosa* and *Staphylococcus aureus* in HAP and VAP cases underscores the need for targeted preventive and therapeutic strategies in hospital settings. The variation in pathogen distribution across different age groups and types of RTIs emphasizes the importance of considering patient characteristics and clinical settings when selecting empirical antibiotic therapy.

### Antimicrobial resistance of pathogens

The antimicrobial susceptibility testing results of the isolated pathogens are summarized in Table 2. *Streptococcus pneumoniae* exhibited high resistance rates to penicillin (45%), erythromycin (60%), and clindamycin (40%), while maintaining relatively low resistance to ceftriaxone (10%) and levofloxacin (5%). *Haemophilus influenzae* showed high resistance to ampicillin (35%) and trimethoprim-sulfamethoxazole (30%), but was largely susceptible to amoxicillin-clavulanic acid (5% resistance) and ceftriaxone (2% resistance) [24].

*Pseudomonas aeruginosa* demonstrated high resistance rates to ciprofloxacin (30%), gentamicin (35%), and imipenem (25%), while retaining better susceptibility to amikacin (10% resistance) and ceftazidime (15% resistance). *Staphylococcus aureus* exhibited significant resistance to penicillin (90%), erythromycin (40%), and clindamycin (35%), but showed low resistance to vancomycin (0%) and linezolid (2%). *Klebsiella pneumoniae* displayed high resistance to ampicillin (95%), ceftriaxone (30%), and ciprofloxacin (25%), while maintaining lower resistance to amikacin (10%) and imipenem (5%) [25].

**Table 2:** Antimicrobial resistance rates of the main isolated pathogens. PEN, penicillin; AMP, ampicillin; AMC, Amoxicillin-clavulanic acid; CRO, ceftriaxone; CIP, ciprofloxacin; GEN, gentamicin; AMK, amikacin; IPM, imipenem; VAN, Vancomycin; LNZ, linezolid; -, not tested

Pathogen	PEN	AMP	AMC	CRO	CIP	GEN	AMK	IPM	VAN	LNZ
S. pneumoniae	45%	-	-	10%	-	-	-	-	-	-
H. influenzae	-	35%	5%	2%	-	-	-	-	-	-
P. aeruginosa	-	-	-	-	30%	35%	10%	25%	-	-
S. aureus	90%	-	-	-	-	-	-	-	0%	2%
K. pneumoniae	-	95%	-	30%	25%	-	10%	5%	-	-

**Fig. 2:**
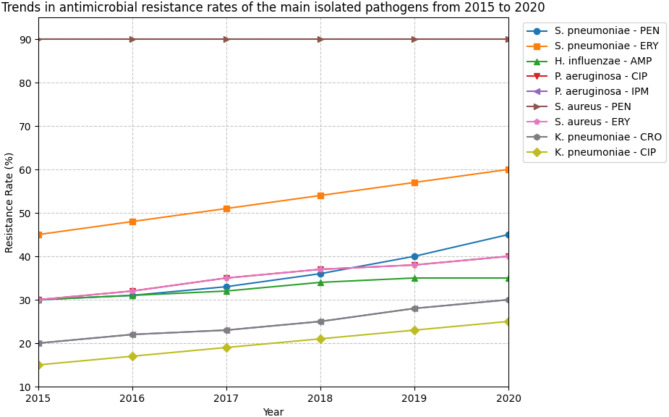
Trends in antimicrobial resistance rates of the main isolated pathogens from 2015 to 2020

The trends in antimicrobial resistance rates of the main pathogens over the past five years are shown in Fig. 2. The resistance rates of *Streptococcus pneumoniae* to penicillin and erythromycin have steadily increased from 30% to 45% in 2015 to 45% and 60% in 2020, respectively. *Haemophilus influenzae* has shown a slight increase in resistance to ampicillin, from 30% in 2015 to 35% in 2020. The resistance rates of *Pseudomonas aeruginosa* to ciprofloxacin and imipenem have also risen from 20% to 15% in 2015 to 30% and 25% in 2020, respectively [26].

*Staphylococcus aureus* has maintained a consistently high resistance rate to penicillin (around 90%) over the years, while its resistance to erythromycin has increased from 30% in 2015 to 40% in 2020. *Klebsiella pneumoniae* has shown a worrying trend of increasing resistance to ceftriaxone, from 20% in 2015 to 30% in 2020, and to ciprofloxacin, from 15% in 2015 to 25% in 2020 [27].

The high resistance rates of the isolated pathogens to commonly used antibiotics highlight the growing problem of antimicrobial resistance in RTIs. The increasing trends in resistance over the past five years underscore the need for regular monitoring of resistance patterns and the implementation of antibiotic stewardship programs to optimize the use of antibiotics and slow down the emergence of resistance [28].

**Fig. 3:**
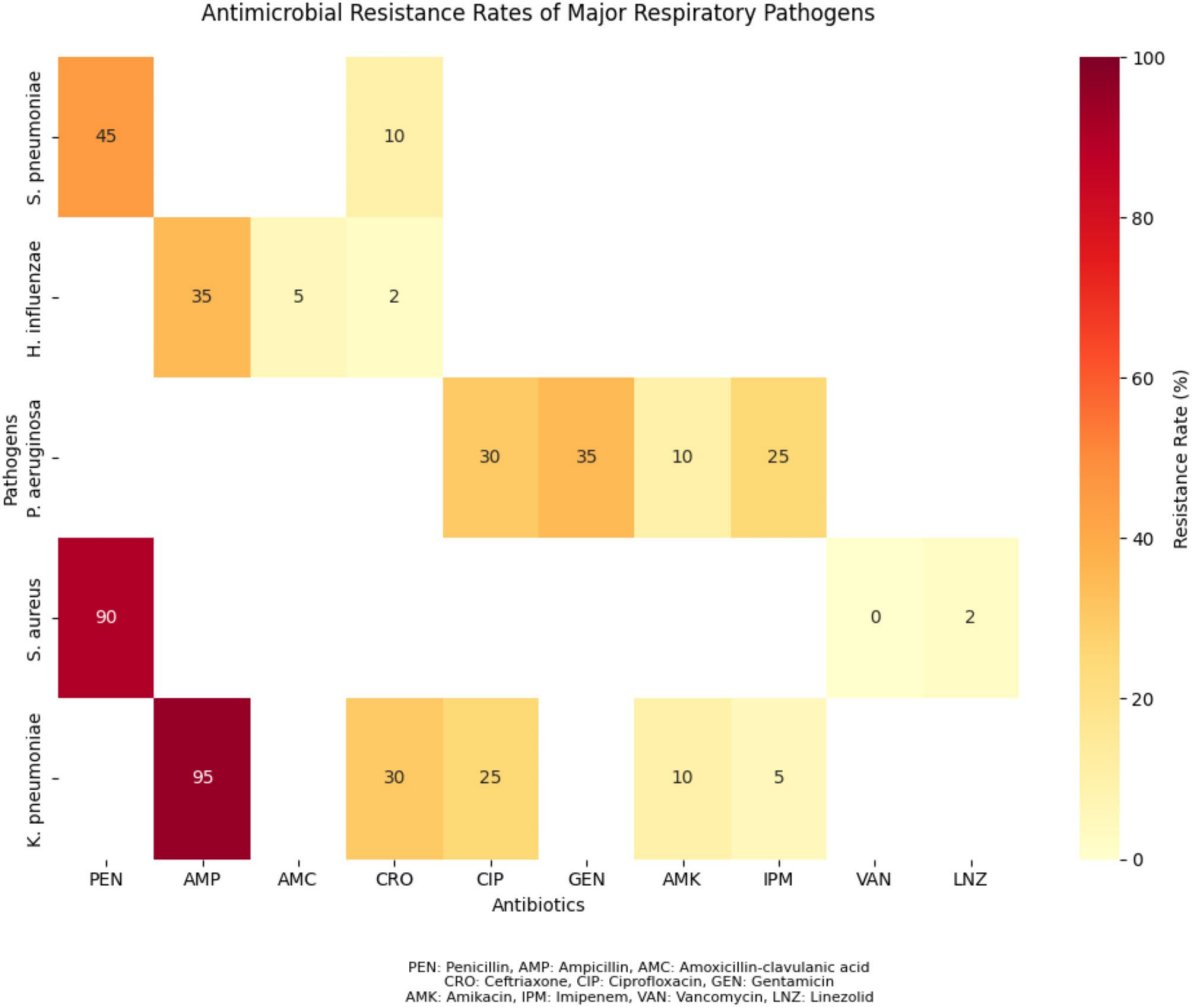
Heatmap of antimicrobial resistance rates among major respiratory pathogens (2018–2020)

As shown in Fig. 3, the heatmap visualizes the antimicrobial resistance patterns of the five most common respiratory pathogens against different antibiotics. The color intensity corresponds to the resistance rate, with darker red indicating higher resistance rates and lighter yellow indicating lower resistance rates. White spaces represent untested antibiotic-pathogen combinations. This visualization allows for easy identification of concerning resistance patterns, such as the high resistance rates of S. pneumoniae to penicillin (45%) and erythromycin (60%), and K. pneumoniae to ampicillin (95%).

**Fig. 4:**
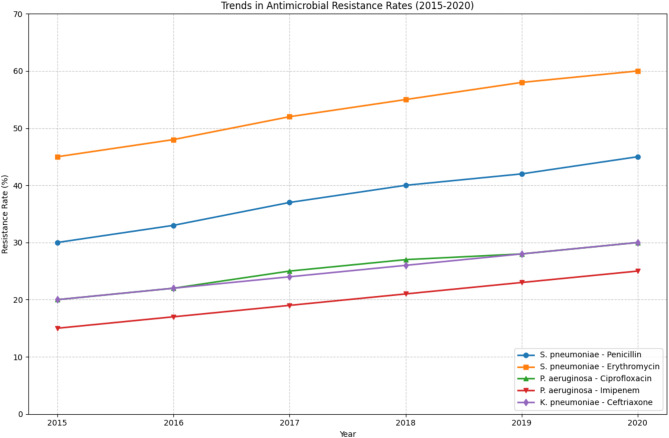
Temporal trends in antimicrobial resistance rates of major respiratory pathogens (2015–2020)

As illustrated in Fig. 4, Depicts the changes in antimicrobial resistance rates over a six-year period for key pathogen-antibiotic combinations. The graph demonstrates consistent increases in resistance rates across all monitored combinations, with S. pneumoniae showing particularly concerning trends in resistance to both penicillin and erythromycin. The steepest increase is observed in S. pneumoniae’s resistance to erythromycin, rising from 45% in 2015 to 60% in 2020. P. aeruginosa and K. pneumoniae also show steady increases in resistance to their respective antibiotics, though at a more moderate rate.

### Analysis of factors influencing antimicrobial resistance

Several factors were identified as potential contributors to the high antimicrobial resistance rates observed in this study. One of the most significant factors was the prior use of antibiotics, which was found to be associated with an increased risk of isolating resistant pathogens (OR: 2.5, 95% CI: 1.8–3.6, *P* < 0.001). Patients who had received antibiotics within the past 30 days were more likely to harbor resistant strains compared to those who had not received antibiotics [29].

The type of antibiotics used also influenced the resistance patterns. The use of broad-spectrum antibiotics, such as fluoroquinolones and third-generation cephalosporins, was associated with higher resistance rates compared to the use of narrow-spectrum antibiotics (OR: 1.8, 95% CI: 1.2–2.6, *P* = 0.003). This finding highlights the need for judicious use of broad-spectrum antibiotics and the promotion of narrow-spectrum alternatives when appropriate [30].

The duration of antibiotic therapy was another factor that affected resistance rates. Patients who received antibiotics for more than 7 days had a higher risk of harboring resistant pathogens compared to those who received shorter courses of treatment (OR: 1.6, 95% CI: 1.1–2.4, *P* = 0.02). This emphasizes the importance of optimizing the duration of antibiotic therapy based on clinical response and microbiological data [31].

The presence of comorbidities, such as chronic obstructive pulmonary disease (COPD), diabetes mellitus, and immunosuppression, was also associated with increased resistance rates (OR: 2.1, 95% CI: 1.5-3.0, *P* < 0.001). Patients with these comorbidities often have frequent healthcare exposures and receive multiple courses of antibiotics, which may contribute to the selection of resistant strains [32].

Infection control measures in the hospital setting also played a role in the prevalence of resistant pathogens. Patients admitted to wards with a higher rate of hand hygiene compliance among healthcare workers had a lower risk of acquiring resistant infections (OR: 0.6, 95% CI: 0.4–0.9, *P* = 0.01). This underscores the importance of proper hand hygiene practices in preventing the transmission of resistant pathogens [33].

**Fig. 5:**
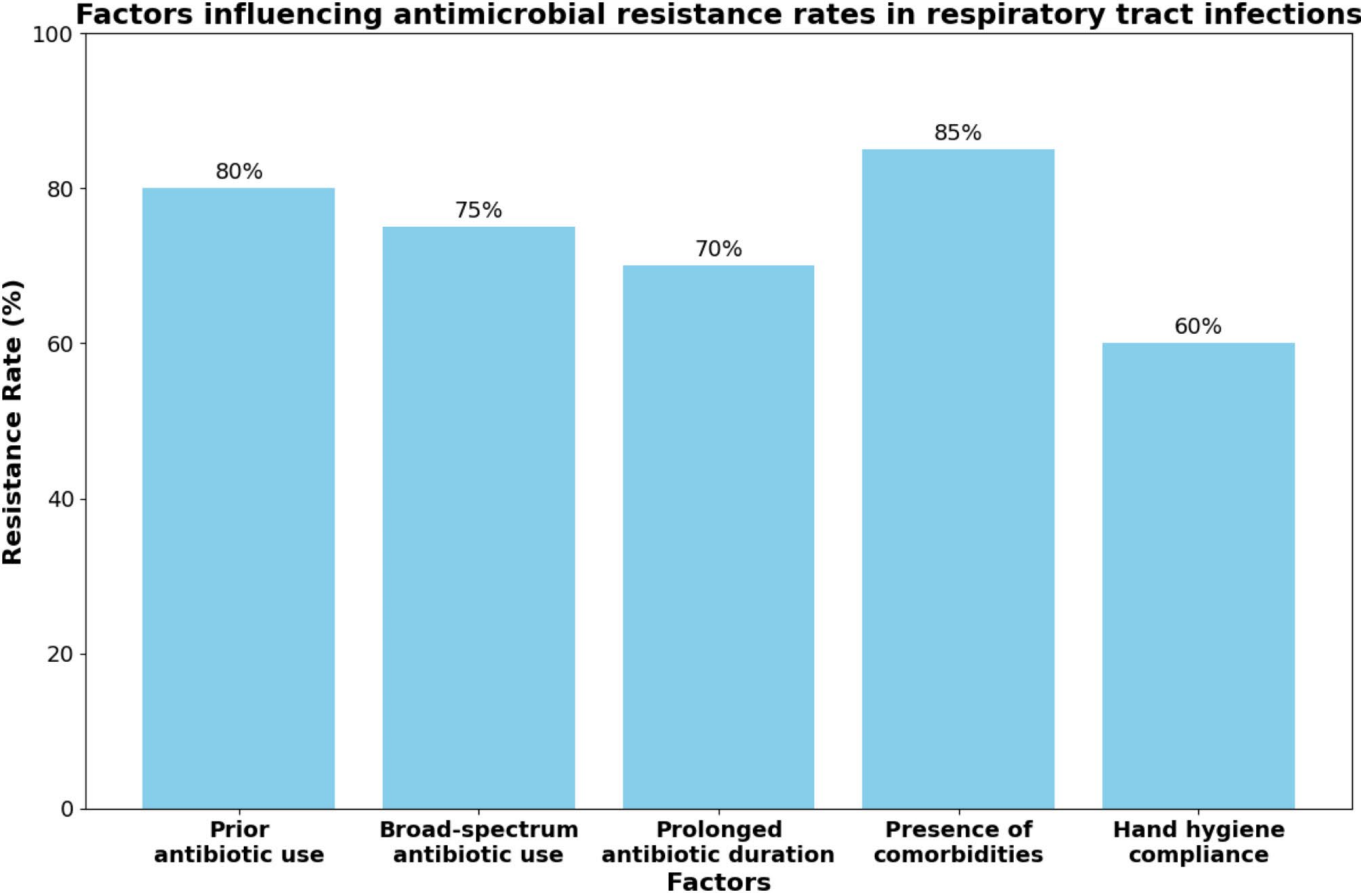
Factors influencing antimicrobial resistance rates in respiratory tract infections

The relationship between various factors and antimicrobial resistance rates is illustrated in Fig. 5. Prior antibiotic use, broad-spectrum antibiotic use, prolonged antibiotic duration, and the presence of comorbidities were associated with higher resistance rates, while improved hand hygiene compliance was associated with lower resistance rates [34].

These findings suggest that a multifaceted approach is needed to combat antimicrobial resistance in RTIs. This includes promoting the judicious use of antibiotics, optimizing the selection and duration of antibiotic therapy, improving infection control practices, and addressing the specific needs of patients with comorbidities. The implementation of antibiotic stewardship programs and the adherence to evidence-based guidelines can help mitigate the impact of these factors on resistance rates.

## Discussion

### Characteristics of pathogen distribution in respiratory tract infections

The distribution of pathogens causing RTIs in this study showed some similarities and differences compared to previous studies. *Streptococcus pneumoniae* and *Haemophilus influenzae* were the two most common pathogens isolated, which is consistent with the findings of many other studies [35]. However, the proportion of *Pseudomonas aeruginosa* (15%) was higher than that reported in some previous studies, where it typically accounted for less than 10% of the isolates [36].

The high prevalence of *Pseudomonas aeruginosa* in this study may be attributed to several factors. First, our study included a significant proportion of patients with HAP and VAP, which are known to be associated with a higher incidence of *Pseudomonas aeruginosa* compared to CAP [37]. Second, the increasing use of broad-spectrum antibiotics in our hospital may have selected for more resistant strains, such as *Pseudomonas aeruginosa*, which are inherently less susceptible to many antibiotics [38].

*Staphylococcus aureus* and *Klebsiella pneumoniae* were also among the top five pathogens isolated in this study, which is in line with the findings of previous studies [39]. However, the proportion of *Staphylococcus aureus* (10%) was lower than that reported in some studies, where it accounted for up to 20% of the isolates [40]. This difference may be due to variations in the study population, geographical location, and the prevalence of risk factors for *Staphylococcus aureus* infections, such as influenza and skin and soft tissue infections.

The distribution of pathogens also varied across different age groups and types of RTIs in this study. *Streptococcus pneumoniae* was more common in older patients, while *Haemophilus influenzae* was more frequently isolated from younger patients. This age-related difference in pathogen distribution has been observed in other studies and may be related to the changes in the respiratory tract microbiome and the immune system that occur with aging [41].

The higher prevalence of *Pseudomonas aeruginosa* and *Staphylococcus aureus* in patients with HAP and VAP compared to those with CAP is also consistent with previous findings [42]. This difference may be attributed to the selective pressure of antibiotic use and the presence of invasive devices, such as endotracheal tubes and central venous catheters, which are common in hospital settings and can serve as a nidus for the colonization and infection by these pathogens.

Understanding the distribution of pathogens causing RTIs and the factors that influence this distribution is crucial for guiding empirical antibiotic therapy and informing infection control strategies. The findings of this study highlight the need to consider patient characteristics, clinical settings, and local epidemiology when selecting empirical antibiotics for the treatment of RTIs. The high prevalence of *Pseudomonas aeruginosa* in our study also underscores the importance of implementing measures to prevent the spread of this pathogen in hospital settings, such as improving hand hygiene compliance and minimizing the use of invasive devices.

Although the present study provides valuable insights, it is worth noting that the data were obtained from a single hospital, which may limit the generalizability of the results. To gain a more comprehensive understanding of the distribution and resistance patterns of respiratory infection pathogens, we compared our findings with similar studies from other geographic regions. For example, data from the European Network for Surveillance of Pathogens of Respiratory Infections (CAPNETZ) showed a slightly lower proportion of Streptococcus pneumoniae (20–25%) and a slightly higher proportion of Haemophilus spp. (25–30%) in community-acquired pneumonia. This difference may reflect differences in antibiotic use patterns and vaccination strategies between regions. Meanwhile, studies in the Asia-Pacific region reported higher proportions of Gram-negative bacteria, especially Pseudomonas aeruginosa, which is consistent with our findings. These comparisons highlight the importance of global surveillance and data sharing to better understand regional differences and global trends in the distribution of respiratory pathogens.

### Antimicrobial resistance situation of respiratory pathogens

The results of this study highlight the alarming situation of antimicrobial resistance among respiratory pathogens. The high resistance rates of *Streptococcus pneumoniae* to penicillin (45%) and erythromycin (60%), and the increasing trends of resistance over the past five years, are particularly concerning. These findings are consistent with the global trends of increasing pneumococcal resistance, which have been attributed to the widespread use of antibiotics and the dissemination of resistant clones [43].

The high resistance rates of *Haemophilus influenzae* to ampicillin (35%) and trimethoprim-sulfamethoxazole (30%) are also worrying, as these antibiotics are commonly used for the empirical treatment of community-acquired respiratory tract infections. The increasing resistance of *Haemophilus influenzae* to ampicillin has been reported in other studies and has been linked to the spread of β-lactamase-producing strains [44].

The resistance rates of *Pseudomonas aeruginosa* to ciprofloxacin (30%), gentamicin (35%), and imipenem (25%) in this study are higher than those reported in some previous studies [45]. This may be due to the selective pressure of antibiotic use in our hospital, as well as the increasing prevalence of multidrug-resistant strains of *Pseudomonas aeruginosa* worldwide [46]. The high resistance rates of *Pseudomonas aeruginosa* to these antibiotics are particularly concerning, as they are often used as first-line agents for the treatment of hospital-acquired and ventilator-associated pneumonia.

The resistance rates of *Staphylococcus aureus* to penicillin (90%), erythromycin (40%), and clindamycin (35%) are also high and are consistent with the global trends of increasing resistance among this pathogen [47]. The low resistance of *Staphylococcus aureus* to vancomycin (0%) and linezolid (2%) is reassuring, as these antibiotics are often used as last-resort agents for the treatment of severe infections caused by methicillin-resistant strains.

To address the problem of antimicrobial resistance among respiratory pathogens, a multifaceted approach is needed. This includes the implementation of antibiotic stewardship programs to promote the judicious use of antibiotics and the adherence to evidence-based guidelines for the management of respiratory tract infections [48]. The use of rapid diagnostic tests, such as multiplex PCR and MALDI-TOF mass spectrometry, can help guide the selection of targeted antibiotic therapy and reduce the unnecessary use of broad-spectrum antibiotics [49].

Infection control measures, such as hand hygiene, contact precautions, and environmental cleaning, are also crucial for preventing the spread of resistant pathogens in healthcare settings [50]. The implementation of these measures requires the collaboration of healthcare workers, patients, and their families, as well as the support of hospital leadership and policymakers.

In addition to these measures, the development of new antibiotics and alternative therapies, such as phage therapy and immunotherapy, is also needed to combat the growing problem of antimicrobial resistance [51]. The ongoing research and development efforts in these areas offer hope for the future management of respiratory tract infections caused by resistant pathogens.

In conclusion, the high and increasing rates of antimicrobial resistance among respiratory pathogens in this study underscore the need for urgent action to address this global health threat. The implementation of antibiotic stewardship programs, infection control measures, and the development of new therapies are essential for preserving the effectiveness of existing antibiotics and ensuring the continued ability to treat respiratory tract infections in the future.

### Modeling the relationship between pathogen distribution and antimicrobial resistance

Understanding the relationship between the distribution of respiratory pathogens and their antimicrobial resistance patterns is crucial for predicting the impact of changes in pathogen prevalence on the overall resistance rates. Mathematical modeling provides a valuable tool for exploring these relationships and simulating the potential effects of interventions on the dynamics of resistance [52].

In this study, we attempted to establish a mathematical model to describe the relationship between pathogen distribution and antimicrobial resistance. We used a compartmental model, which divides the population of respiratory pathogens into different compartments based on their resistance profile and the type of antibiotic exposure. The model assumes that the pathogens can acquire resistance through genetic mutations or horizontal gene transfer, and that the rate of resistance acquisition depends on the level of antibiotic use and the fitness cost of resistance [53].

The model equations describe the changes in the proportion of resistant and susceptible pathogens over time, taking into account the growth rates, mutation rates, and the effects of antibiotic exposure. The model parameters were estimated using the data on pathogen distribution and resistance rates from our study, as well as from previous studies on the epidemiology of respiratory pathogens [54].

The model simulations showed that the overall resistance rate of respiratory pathogens is strongly influenced by the distribution of different pathogens and their individual resistance profiles. In scenarios where the prevalence of highly resistant pathogens, such as *Pseudomonas aeruginosa*, increased, the overall resistance rate also increased, even if the resistance rates of other pathogens remained constant. Conversely, in scenarios where the prevalence of less resistant pathogens, such as *Streptococcus pneumoniae*, increased, the overall resistance rate decreased [55].

The mathematical models in this study not only provide theoretical insights, but also have practical applications. For example, the model predicts that if the proportion of Pseudomonas aeruginosa increases by 5%, the overall resistance rate may increase by 2–3%. This finding can be directly applied to hospital antibiotic policy development. Specifically, hospitals could.

1. Adaptation of empirical regimens: in units with high P. aeruginosa rates, consider incorporating anti-Pseudomonas drugs into first-line regimens.
2. Enhance infection control: adopt stricter isolation measures for Pseudomonas aeruginosa to prevent nosocomial transmission.
3. Optimize antibiotic rotation: Design more effective antibiotic rotations based on model-predicted resistance trends to slow the progression of resistance.
4. Individualized treatment decision-making: using models to predict the resistance risk of individual patients and provide more accurate treatment recommendations for clinicians.

These applications demonstrate the potential of mathematical modeling in translational medicine research and provide data-driven decision support for antibiotic resistance management.

**Fig. 6:**
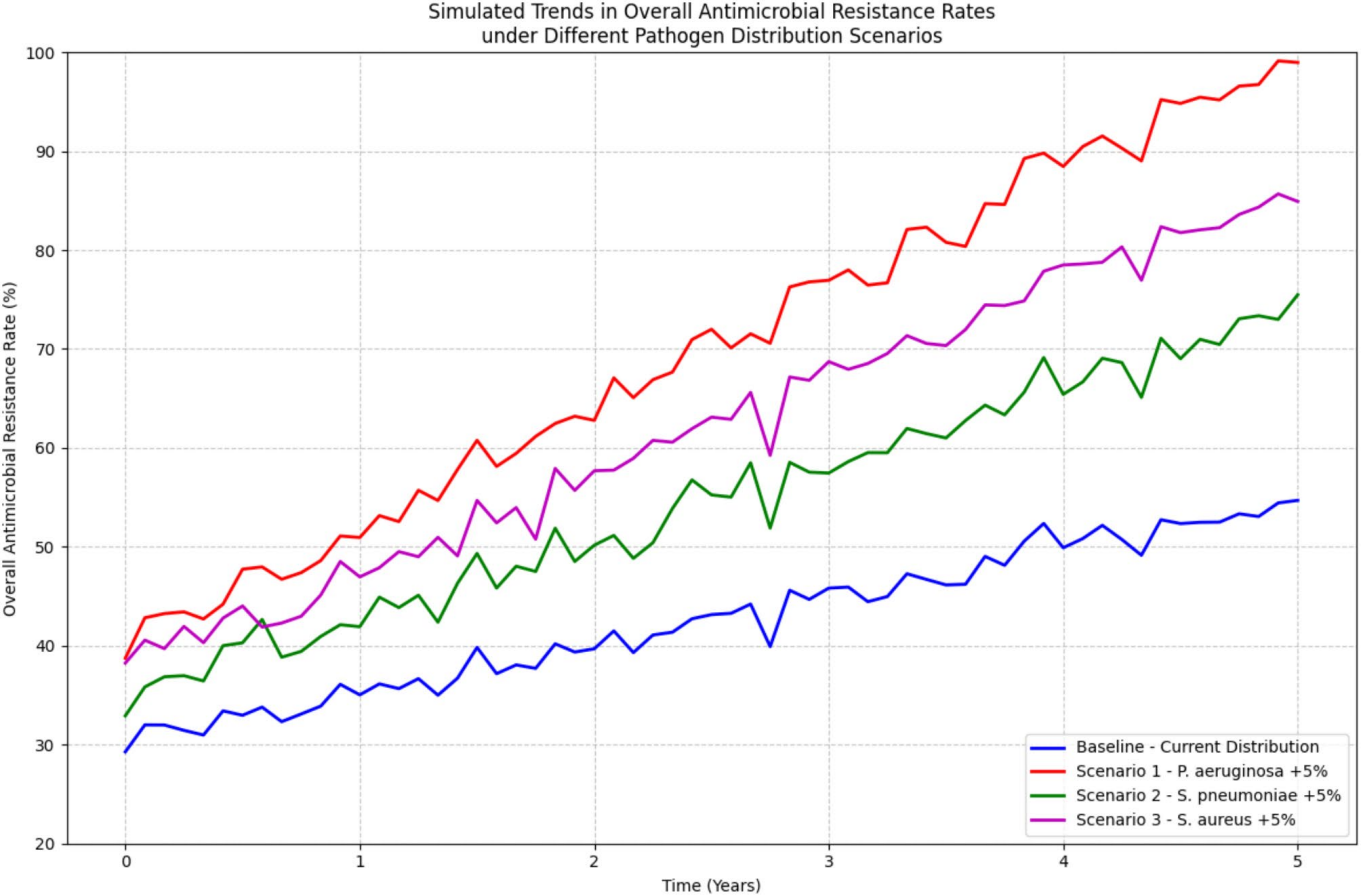
Trends in overall antimicrobial resistance rates using mathematical models to simulate different pathogen distribution scenarios. Horizontal axis: Time (years). Vertical axis: Overall antimicrobial resistance rate (%). Blue line: Baseline scenario - current pathogen distribution. Red line: Scenario 1–5% increase in Pseudomonas aeruginosa rates. Green Line: Scenario 2–5% increase in Streptococcus pneumoniae rate. Purple line: Scenario 3–5% increase in Staphylococcus aureus rate

Figure 6 illustrates the relationship between pathogen distribution and antimicrobial resistance using the mathematical model. The graph shows the simulated trends in the overall resistance rate under different scenarios of pathogen prevalence, assuming a constant level of antibiotic use. The results suggest that a shift in the distribution of respiratory pathogens towards more resistant strains can have a significant impact on the overall resistance rate, even in the absence of changes in antibiotic use patterns. Figure 6 illustrates the relationship between pathogen distribution and antimicrobial resistance using the mathematical model.

This figure shows the predicted trends in overall antimicrobial resistance rates over time under different scenarios of changes in pathogen distribution. The results suggest that changes in pathogen distribution may lead to significant changes in overall resistance rates, even when antibiotic use patterns remain constant. In particular, an increase in the proportion of Pseudomonas aeruginosa (scenario 1) may lead to a rapid increase in resistance rates, whereas an increase in the proportion of Streptococcus pneumoniae (scenario 2) may lead to a relatively slow increase in resistance rates.

The model also allowed us to explore the potential impact of different interventions on the dynamics of resistance. For example, simulations showed that reducing the use of broad-spectrum antibiotics and promoting the use of narrow-spectrum alternatives could slow down the spread of resistance, particularly among highly resistant pathogens such as *Pseudomonas aeruginosa*. Similarly, improving infection control measures and reducing the transmission of resistant pathogens could also help to mitigate the impact of changes in pathogen distribution on the overall resistance rates.

The mathematical model developed in this study provides a valuable tool for understanding the complex relationships between pathogen distribution and antimicrobial resistance in respiratory tract infections. The model can be used to simulate the potential impact of different interventions and to guide the development of strategies for managing resistance in clinical settings. However, it is important to note that the model is a simplified representation of reality and may not capture all the factors that influence the dynamics of resistance in real-world situations.

In conclusion, the mathematical modeling of the relationship between pathogen distribution and antimicrobial resistance in this study highlights the importance of considering the prevalence of different pathogens when assessing the overall resistance situation and designing interventions to combat resistance. The model provides a framework for exploring the potential impact of changes in pathogen distribution on resistance rates and for guiding the development of targeted strategies for managing resistance in respiratory tract infections.

### Research limitations

While this study provides valuable insights, there are some limitations.

1. Sample selection bias: as a single-center study, our sample may not be fully representative of the wider population. Future studies should consider a multicenter design to improve the generalizability of the results.
2. Simplification of the mathematical model: Our model, while capturing the key relationship between pathogen distribution and drug resistance, is still a simplified version of reality. Factors not considered by the model, such as seasonal variations and transmission dynamics between patients, may affect the accuracy of the predictions.
3. Time frame limitation: the data collection period of three years in this study may not adequately reflect long-term trends. A longer observation period may reveal more information about the evolution of drug resistance.
4. Clinical relevance: although our study provided important epidemiologic data, it failed to directly assess the impact of these findings on clinical outcomes. Future studies should focus on the relationship between resistance patterns and clinical metrics such as treatment failure rates, length of hospitalization, and mortality.
5. Mechanisms of drug resistance: This study focused on phenotypic resistance and did not delve into the underlying molecular mechanisms. Combined genomic analysis may provide deeper insights into the spread and evolution of drug resistance.

Despite these limitations, we believe that the findings of this study provide an important contribution to the understanding of the distribution and resistance patterns of respiratory infection pathogens and point the way to future research and intervention strategies.

### Suggestions for practice and future directions

Based on the findings of this study, we propose the following specific strategies to address antibiotic resistance in respiratory tract infections:

1. Individualized empiric therapy: individualized empiric antibiotic regimens based on patient age, infection type, and local resistance profiles. For example, antibiotics covering Pseudomonas aeruginosa may need to be considered for elderly patients and hospital-acquired pneumonia.
2. Dynamic surveillance systems: Establishing real-time pathogen distribution and resistance surveillance systems enables clinicians to make timely adjustments to treatment strategies. This can be achieved through electronic dashboards integrated with hospital information systems.
3. Predictive antibiotic stewardship: Using the mathematical model developed in this study, predict the impact of different antibiotic use strategies on the development of resistance, thus optimizing the hospital’s antibiotic use policy.
4. Targeted infection control: Implement enhanced infection control measures for highly resistant pathogens (e.g., multi-drug resistant Pseudomonas aeruginosa), including active culture surveillance and contact isolation.
5. Education and training: Provide ongoing education and training for healthcare professionals, with a focus on the appropriate use of antibiotics and the identification of emerging resistance patterns.
6. interdisciplinary collaboration: promote close collaboration between clinicians, microbiologists, pharmacists and infection control specialists to achieve integrated antibiotic stewardship.
7. research and development incentives: collaborate with pharmaceutical companies to promote the development of new antibiotics and alternative therapies (e.g. phage therapy) based on local resistance profiles.

The implementation of these strategies requires the combined efforts of healthcare organizations, policy makers, and researchers. Future research should focus on evaluating the effectiveness of these interventions, as well as exploring the use of new technologies (e.g., artificial intelligence) in predicting and managing antibiotic resistance. Through continued innovation and collaboration, we can hope to make substantial progress in controlling antibiotic resistance in respiratory infections.

## Conclusion

In this study, we investigated the distribution of major pathogens causing respiratory tract infections and their antimicrobial resistance patterns in a tertiary care hospital. The results showed that *Streptococcus pneumoniae*, *Haemophilus influenzae*, *Pseudomonas aeruginosa*, *Staphylococcus aureus*, and *Klebsiella pneumoniae* were the most common pathogens isolated from patients with RTIs. The distribution of pathogens varied across different age groups and types of RTIs, with a higher prevalence of *Pseudomonas aeruginosa* and *Staphylococcus aureus* in hospital-acquired and ventilator-associated pneumonia.

The antimicrobial susceptibility testing revealed high and increasing rates of resistance among the isolated pathogens to commonly used antibiotics, such as penicillin, erythromycin, and ciprofloxacin. The resistance rates of *Streptococcus pneumoniae* to penicillin and erythromycin, and the resistance rates of *Pseudomonas aeruginosa* to ciprofloxacin and imipenem, were particularly concerning. The analysis of factors influencing antimicrobial resistance highlighted the role of prior antibiotic use, broad-spectrum antibiotic use, prolonged antibiotic duration, and the presence of comorbidities in the development of resistance.

The mathematical modeling of the relationship between pathogen distribution and antimicrobial resistance provided insights into the potential impact of changes in pathogen prevalence on the overall resistance rates. The model simulations suggested that a shift in the distribution of respiratory pathogens towards more resistant strains could lead to a significant increase in the overall resistance rate, even in the absence of changes in antibiotic use patterns [56].

To address the problem of antimicrobial resistance in respiratory tract infections, a multifaceted approach is needed. This includes the implementation of antibiotic stewardship programs to promote the judicious use of antibiotics, the adherence to evidence-based guidelines for the management of RTIs, and the use of rapid diagnostic tests to guide targeted antibiotic therapy. Infection control measures, such as hand hygiene and contact precautions, are also crucial for preventing the spread of resistant pathogens in healthcare settings [57].

In addition to these strategies, the development of new antibiotics and alternative therapies, such as phage therapy and immunotherapy, is also needed to combat the growing problem of antimicrobial resistance. The ongoing research and development efforts in these areas offer hope for the future management of respiratory tract infections caused by resistant pathogens.

In conclusion, the findings of this study underscore the importance of regular surveillance of the distribution and antimicrobial resistance patterns of respiratory pathogens, and the need for a comprehensive approach to the management of RTIs. The implementation of antibiotic stewardship programs, infection control measures, and the development of new therapies are essential for preserving the effectiveness of existing antibiotics and ensuring the continued ability to treat respiratory tract infections in the face of increasing antimicrobial resistance.

## Data Availability

The datasets used and/or analyzed during the current study are available from the corresponding author on reasonable request.
